# Montage Error in Ultra–Widefield Imaging of Retinal Hemangioblastomas

**DOI:** 10.1155/crop/3979304

**Published:** 2026-06-23

**Authors:** Mohamed Belmouhand, Jens Folke Kiilgaard, Carsten Faber

**Affiliations:** ^1^ Department of Ophthalmology, Copenhagen University Hospital, Rigshospitalet, Copenhagen, Denmark, gentoftehospital.dk; ^2^ Department of Clinical Medicine, University of Copenhagen, Copenhagen, Denmark, ku.dk

**Keywords:** artifact, retinal hemangioblastoma, ultra–widefield imaging, von Hippel–Lindau

## Abstract

A 20‐year‐old male presented with three large retinal hemangioblastomas in the left eye. At the 5‐week follow‐up, automated montage ultra–widefield imaging appeared to demonstrate a fourth lesion, suggesting possible disease progression. Clinical examination, however, confirmed the presence of only three tumors. Re‐evaluation of the automated image stitching revealed misidentification of the optic nerve head in one of the source images, resulting in image misalignment and the false appearance of an additional lesion. Systemic evaluation demonstrated central nervous system hemangioblastomas and a renal cyst, and genetic testing confirmed von Hippel–Lindau disease. This case illustrates both the diagnostic utility and limitations of ultra–widefield montage imaging and highlights the importance of clinical verification of apparent lesion increase before concluding true progression.

## 1. Introduction

Retinal hemangioblastomas (RHs) are benign vascular tumors that may occur sporadically or in association with von Hippel–Lindau (VHL) disease. Early identification is clinically important, as ocular findings may represent the first manifestation of a multisystem disorder [1]. Ultra–widefield (UWF) imaging has become an integral tool in ophthalmology, particularly within ocular oncology, enabling visualization of the peripheral retina beyond conventional imaging fields and facilitating documentation, monitoring, and treatment planning [2–4]. However, UWF imaging is susceptible to artifacts related to image acquisition and postprocessing, including misregistration and geometric distortion, which may complicate interpretation [5, 6].

## 2. Case Report

A 20‐year‐old previously healthy male was referred with a presumed diagnosis of retinal detachment in the left eye. The patient reported a gradual decrease in vision in the left eye over several years, with recent worsening including a curtain‐like shadow during the week prior to presentation. Best‐corrected visual acuity was Snellen 1.0 in the right eye and 0.07 in the left eye. Refraction was −1.5 spherical diopters bilaterally. Intraocular pressure measured by handheld rebound tonometry was 17 mmHg in the right eye and 14 mmHg in the left eye. Both eyes were white and quiet, and the anterior segments were deep and unremarkable.

**Figure 1:**
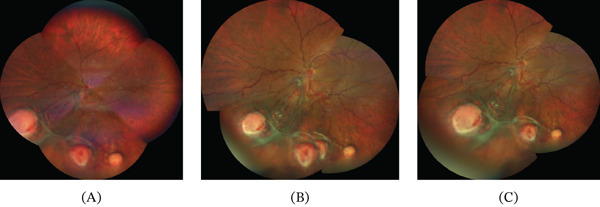
Ultra–widefield imaging of the left eye. (A) Baseline image showing three large RHs. (B) The 5‐week follow‐up image falsely suggesting an additional lesion due to a stitching artifact during automated montage generation. (C) Repeat montage generation with manual identification of the optic nerve head corrected the major alignment error and eliminated the false appearance of an additional lesion. Minor vascular misalignment in the inferior quadrants remained visible.

Fundoscopy of the right eye was unremarkable. Fundoscopy of the left eye revealed a moderate vitreal reaction, a dense posterior hyaloid, and three large RHs located in the inferior peripheral retina with prominent feeder vessels. The largest RH measured 2.6 mm in thickness on B‐scan ultrasonography. UWF fluorescein angiography demonstrated early hyperfluorescence with late‐phase staining of the three large RHs, as well as two small RHs (< 1 disc diameter) in the right eye and five similarly sized RHs in the left eye. The lesions were documented under dilatation using automated montage UWF imaging with the Zeiss Clarus 700 fundus camera (Figure 1A). The patient was scheduled for laser photocoagulation of the small RHs and ruthenium‐106 brachytherapy of the three large RHs.

At the 5‐week follow‐up prior to treatment, repeat dilated fundus imaging using the same technique appeared to demonstrate four large RHs in the left eye (Figure 1B). Clinical examination, however, confirmed the presence of only three lesions. This finding was further supported by re‐evaluation of the four individual images used to generate the montage. Subsequent reassessment of the automated image stitching revealed incorrect identification of the optic nerve head in one of the images, resulting in image misalignment. Repeat montage generation with manual identification of the optic nerve head produced correct alignment of the four images and eliminated the false appearance of an additional lesion. However, residual misalignment of retinal vessels in the inferior quadrants remained visible (Figure 1C).

Systemic evaluation with MRI demonstrated hemangioblastomas in the cerebellum and along the spinal cord, as well as a large cyst in the right kidney. Genetic testing confirmed a VHL gene mutation, establishing the diagnosis of VHL disease. Although a solitary RH carries an estimated 46% probability of underlying VHL disease, decreasing with age [7], the presence of two or more lesions fulfills clinical diagnostic criteria for VHL disease [8].

## 3. Discussion

This case illustrates a clinically relevant limitation of montage‐based UWF imaging. Misregistration between sequentially acquired frames resulted in a stitching artifact that simulated an additional RH. In patients with VHL, lesion number is clinically important for both diagnosis and longitudinal surveillance, and false lesion duplication may therefore lead to overestimation of disease burden or apparent progression.

The novelty of this report lies in demonstrating that incorrect identification of the optic nerve head during automated image stitching can produce false lesion duplication and apparent disease progression during longitudinal follow‐up. As the Zeiss Clarus system relies on the optic nerve head as a key landmark for image registration, accurate identification is essential for reliable montage construction. In this case, incorrect optic nerve head registration caused image misalignment despite otherwise high image quality and absence of obvious global distortion. This finding is relevant not only to VHL but also more broadly to other more common retinal and choroidal disorders monitored with UWF imaging.

Previous studies have demonstrated horizontal distortion [6], as well as peripheral optical and projection‐related aberrations in UWF imaging [2, 5]. These limitations are particularly relevant during longitudinal assessment and teleophthalmology‐based evaluation, where subtle artifacts may influence interpretation of lesion number, size, or progression. As UWF imaging becomes increasingly integrated into clinical practice, awareness of potential image artifacts is essential. Critical evaluation of images and correlation with clinical examination remain important, particularly when new lesions or disease progression are suspected based on imaging findings alone.

## Author Contributions

Data interpretation: M.B., J.F.K., and C.F. Manuscript drafting: C.F. Critical revision and final approval of the manuscript: M.B., J.F.K., and C.F.

## Funding

No funding was received for this manuscript.

## Disclosure

The authors have nothing to report.

## Ethics Statement

The patient provided informed consent for publication of this case, including anonymized ocular images and associated systemic and genetic findings.

## Conflicts of Interest

The authors declare no conflicts of interest.

## Data Availability

The data that support the findings of this study are available from the corresponding author upon reasonable request.

## References

[1] Singh A. D. , Shields C. L. , and Shields J. A. , von Hippel–Lindau Disease, Survey of Ophthalmology. (2001) 46, no. 2, 117–142, 10.1016/S0039-6257(01)00245-4. 11578646

[2] Callaway N. F. and Mruthyunjaya P. , Widefield Imaging of Retinal and Choroidal Tumors, International Journal of Retina and Vitreous. (2019) 5, no. Supplement 1, 10.1186/s40942-019-0196-5, 31890289. PMC6907111 31890289

[3] Lucente A. , Taloni A. , Scorcia V. , and Giannaccare G. , Widefield and Ultra-Widefield Retinal Imaging: A Geometrical Analysis, Life. (2023) 13, no. 1, 10.3390/life13010202, 36676151. PMC9867331 36676151

[4] Nagiel A. , Lalane R. A. , Sadda S. R. , and Schwartz S. D. , Ultra-Widefield Fundus Imaging: A Review of Clinical Applications and Future Trends, Retina. (2016) 36, no. 4, 660–678, 10.1097/IAE.0000000000000937. 27014860

[5] Huang B. , Zheng C. , Chen S. , Liao X. , and Chen H. , Quantifying Retinal Size and Shape Distortion in Different Ultra-Widefield Imaging Systems, BMJ Open Ophthalmology. (2025) 10, no. 1, e001965, 10.1136/bmjophth-2024-001965, 40154565. PMC11956332 40154565

[6] Muttuvelu D. V. and Kiilgaard J. F. , Inconsistent Distortion in Ultra-Widefield Fundus Image, Acta Ophthalmologica. (2019) 97, no. 2, e326–e327, 10.1111/aos.13951, 30318783. 30318783

[7] Singh A. , Shields J. , and Shields C. , Solitary Retinal Capillary Hemangioma: Hereditary (von Hippel-Lindau Disease) or Nonhereditary?, Archives of Ophthalmology. (2001) 119, no. 2, 232–234, 10.1001/archopht.119.2.232. 11176984

[8] Binderup M. L. M. , Smerdel M. , Borgwadt L. , Nielsen S. S. B. , Madsen M. G. , Møller H. U. , Kiilgaard J. F. , Friis-Hansen L. , Harbud V. , Cortnum S. , and Owen H. , von Hippel-Lindau Disease: Updated Guideline for Diagnosis and Surveillance, European Journal of Medical Genetics. (2022) 65, no. 8, 104538, 10.1016/j.ejmg.2022.104538, 35709961. 35709961

